# Schizophasia and Cognitive Impairment in Schizophrenia: A Literature Review

**DOI:** 10.3390/brainsci15010025

**Published:** 2024-12-29

**Authors:** Sylwia Niedźwiadek, Agata Szulc

**Affiliations:** Department of Psychiatry, Faculty of Health Sciences, Medical University of Warsaw, Partyzantów 2/4, 05-802 Pruszkow, Poland

**Keywords:** linguistic, cognitive, schizophrenia, schizophasia

## Abstract

Background: Cognitive functions are the basis for the development of language skills. Cognitive disorders occur in schizophrenia and may be present even before the first symptoms of psychosis. Language deficits are also mentioned as one of the diagnostic symptoms of this disease. Methods: A literature search was performed using the PubMed database. Articles comparing linguistic and cognitive functioning in schizophrenia were searched for. Following the inclusion and exclusion criteria, twenty-six original articles were selected. Results: Most studies have observed a partial correlation between language and cognitive deficits. The correlation most often involved some cognitive functions or some components of language assessment. Conclusions: The correlation reported in many studies shows that there is a correlation between language and cognitive deficits in schizophrenia. However, numerous studies contradict these reports. It is, therefore, possible that this correlation exists, but not in all patients. Future research should therefore be aimed at identifying in which patients this correlation is present.

## 1. Introduction

Cognition is defined as the particular ability of an individual to perceive information from the environment and process it in order to control their own actions and adapt to environmental conditions. In cognitive psychology, mental processes such as memory, thinking, or imagination are understood as phases of information processing [1]. The speech therapy literature points to a specific relationship between cognition and language, providing a definition of speech as “the set of activities that a person performs with the help of language to learn about the world and to communicate their interpretation to other participants in social life” [2] (p. 15). This definition of speech therefore refers not only to linguistic, communicative, and socialising activities but also to cognitive activities. In cognitive psychology, special attention is paid to the organisation of the cognitive representation of the world, conceived as an actively and independently created construction by the mind, reflecting the surrounding reality [1]. Linguists emphasise that cognitive activities are possible due to language, as it is the language that enables the construction of a mental representation of reality through conceptually organised knowledge [2].

The very first clinical reports of the illness, now referred to as schizophrenia, include mentions of disorganised thinking (speech). Kraepelin, who used the term dementia praecox in reference to the illness, described akataphasia, i.e., inability to find the appropriate expression for a thought. In describing the fundamental symptoms of schizophrenia, Bleuler pointed to the loosening of association as the cause of formal thought disorder (FTD) [3]. The term FTD invariably functions in psychiatry to this day and is used to report the clinical symptoms of this illness. Multiple attempts to define the term have produced findings of a specific relationship between disorganised thinking and speech. Assessment of thought processes is only possible on the basis of the patient’s speech, so when speaking of FTD, clinicians in practice mean speech disorders [4]. Comparing it with FTD, considered as one of the axial symptoms of schizophrenia, language impairment must also be regarded as one of the critical symptoms of this illness.

Indeed, as described in the first paragraph, linguistic functioning is underpinned by cognitive skills, and it may, therefore, be particularly interesting to explore the relationship between linguistic and cognitive functioning in people with schizophrenia. Considering that cognitive deficits are globally impaired even in people at very high risk of psychosis [5,6,7,8], assessing the extent to which they are associated with language deficits may have clinical applications in predicting the onset of the illness.

In this review, we would like to present the current state of knowledge about the co-occurrence of language and cognitive deficits in people with schizophrenia. We expect that there is a correlation between the level of linguistic and cognitive functioning among this group of patients.

## 2. Schizophasia

Disorganised speech (thought disorder) is not only a characteristic but also a diagnostic symptom of schizophrenia [9]. Although the DSM-5 (American Psychiatric Association—APA) emphasises that mild language symptoms are common and non-specific and that only symptoms interfering with effective communication are considered psychopathological, it also draws attention to the considerable difficulty in assessing the severity of language symptoms [9]. While the ICD-11 (World Health Organization—WHO) lists disorganised thinking among the diagnostic symptoms, it points to language symptoms when providing examples of this disorganisation [10]. One of the best-known clinical scales for assessing language pathology was developed by Andreasen—the Scale for the Assessment of Thought, Language and Communication (TLC) [11]. This scale consists of eighteen linguistic phenomena, the most common of which, according to Czernikiewicz [4], are poverty of content of speech, derailment, illogicality, and loss of goal. Linguists use the term ‘schizophasia’ to describe a speech disorder occurring in the course of schizophrenia. Schizophasia is defined as a loss of coherence of speech at the pragmatic, semantic, formal, and grammatical levels [12], which is related, in turn, to the ability to construct utterances in such a way as to effectively realise one’s intentions (so that they are comprehensible to the recipient), consistency in naming and interpreting reality, and correct use of the grammatical rules of a given language. Studies conducted by linguists highlight that in the utterances of patients who have schizophrenia, the number of simple sentences is increased compared to other types of utterances. In addition, a significant reduction in subordinate complex sentences is noted [13]. English-speaking researchers also indicate that the utterances of patients with schizophrenia are characterised by lower syntactic complexity [14]. Research conducted by Woźniak [13] shows that in schizophrenia, the relationship between the name and its meaning is disintegrated. This manifests itself in the construction of utterances based on the similarity of sounds (in the TLC scale, this phenomenon is called clanging), the attribution of meanings to names while omitting relations of similarity and belonging, and the creation of incomprehensible forms—idiolectal neologisms (not motivated by word formation)—which are an original feature of language in schizophrenia. The disintegration of the links between names and meanings indicates disturbances in the mind’s conceptually organised cognitive representations. Clinical studies assessing the linguistic functioning of patients with schizophrenia conducted by Spanish-speaking, Italian-speaking, Japanese-speaking, Brazilian Portuguese-speaking, and German-speaking researchers, among others, have presented analogous findings regarding linguistic deficits in pragmatic [15,16], semantic [15,17], and formal and grammatical [18,19] functioning. A reduction in semantic coherence has also been observed in Indonesian adolescents in the prodromal phase [20].

Linear connotation disorders are also a specific feature of the utterances of patients with schizophrenia. The concept of connotation is understood as the ability, provided by the language system, to connect words to each other [21]. Thus, disorders of linear connotation are unusual combinations of words not appearing in the utterances of other language users [12]. Lipski emphasises that both the disintegration of the linguistic connotative potentials of lexical units and the degree of this disintegration indicate the level of development of the psychotic process. In practice, this means that linear connotation disorders affect patients with a poorer prognosis [21].

According to the view of Woźniak [22], narration is a procedure of interpreting the world with the help of language. In narration, a person demonstrates ways of intellectualising sensory experiences [2]. Thus, they communicate knowledge linguistically, formed in minds as cognitive representations (organised conceptually). Narrative is described as the most difficult form of human linguistic activity [2]. Research on narrative in schizophrenia has led to the conclusion that schizophrenia affects a person’s narrative abilities. Adult patients with a first episode of schizophrenia show the highest narrative coherence, while patients with a very early onset of the illness show reduced narrative coherence. The narrative coherence of patients with chronic illness is significantly impaired. Moreover, in all groups studied, as the complexity of the narrative units increases, their coherence decreases [22].

Some distortions regarding the prosody of speech have been reported in the utterances of patients with schizophrenia [13]. These distortions include the introduction of excessively long pauses, which break up the intonational form of the sentence; the production of utterances on a single tone (intonational flattening); and the filling of the intonational contour of the utterance with meaningless sounds and vocalisations.

The content presented above shows that disorders affecting the language system are observed in patients with schizophrenia. According to the definition of speech provided by Grabias [2], human cognitive activities are performed by means of language. Thomas [23] points out that research on language deficits should be directed at determining in detail at which level of language communication is ‘broken’ and how these disruptions correlate not only with psychopathology but also with cognitive deficits. The aim of this paper is to present findings from the literature addressing linguistic and cognitive functioning in schizophrenia.

## 3. Formal Thought Disorder (FTD)

Changes in language functioning in patients with schizophrenia can affect all levels of language [24]. Czernikiewicz [4] proposes the thesis of a dissolution of the language system. The researcher shows that the uniqueness of linguistic pathology in schizophrenia is constituted by phenomena that disrupt the coherence of speech at its highest textual level. Indeed, linguistic phenomena that interfere with the ability to construct coherent and comprehensible speech are common in chronically ill patients. Several functions are required for this, such as working and short-term memory, planning, monitoring, and control of speech [4]. The WHO identifies cognitive deficits as one of the possible symptoms present in the course of this disease [10,25]. It points out that intellectual performance is usually preserved [25]. Overlapping deficits in working memory, executive function disorders, and slowed reaction time, among other factors, may generate a deterioration in patients’ intellectual performance [26]. IQ test results of 692 men before their first schizophrenic episode and after they were diagnosed with schizophrenia were analysed. Data corresponding to a control group were also extracted. Men who later developed schizophrenia achieved lower scores than members of the control group. In addition, the earlier the patients experienced their first psychotic episode, the lower their scores were. It is also interesting to note that patients’ scores were lower than the mean even long before the first symptoms of the illness occurred [27]. Numerous studies indicate that cognitive deficits may be present even before the first symptoms of psychosis occur [28,29,30,31].

The neurodevelopmental theory developed by Andreasen assumes that there are disturbances in the formation and functioning of neural networks (misconnection syndrome). The causes of these disruptions can include genetic factors, viruses, toxins, or perinatal trauma, which can affect brain development from conception to early adulthood. These result in the phenomenon of cognitive dysmetria, which should be understood as a disturbance in the coordination between thinking and acting, involving malfunction of cognitive processes such as attention, memory, language, or executive functions [32]. According to this theory, the symptoms that show the greatest convergence with cognitive deficits are the negative symptoms of schizophrenia (including apathy, anhedonia, shallow affect, and “poverty of language”). Pathological mechanisms that lead to developmental changes in the brain during foetal life generate damage to basic cognitive processes, leading to psychopathological symptoms typical of schizophrenia [33].

Given that, based on the concept of cognitive dysmetria, a pathogenetic and therapeutic relationship between cognitive deficits and negative symptoms is assumed, and since the primary aim of this work is to indicate possible correlations between cognitive and language deficits in schizophrenia, the question arises: Do disorders of the language system belong to the category of negative symptoms? Andreasen divides the phenomena she has indicated (TLC scale) into those that belong to positive symptoms (e.g., derailment, tangentiality, circumstantiality, illogicality, neologisms, incoherence) and those that belong to negative symptoms (e.g., poverty of content, poverty of speech, blocking) [34,35]. Most of the phenomena, however, were placed in the positive symptom group. Andreasen’s position has been adopted by many researchers, who have also considered language system disorders as part of the positive syndrome of schizophrenia [36]. It is worth mentioning, however, that the two-dimensional model of schizophrenia (dividing its symptoms into positive-productive and negative-deficit symptoms) is not the only proposal for distinguishing the different clinical dimensions of schizophrenia. There are also models indicating three, four, or even five clinical dimensions of this illness [37]. It seems interesting to note that in three-factor models, language disorders are never classified as positive symptoms. Most often, three-factor models include a positive symptom dimension, a negative symptom dimension, and a third dimension related to cognitive impairment. Analyses conducted by Czernikiewicz show that many researchers include FTD precisely in this third dimension [36]. FTD was identified as one of the core symptoms of schizophrenia in 1911 by Bleuler, who popularised the name ‘schizophrenia’ [33,37]. Bleuler considered disintegration to be the disorder that produces the symptoms of schizophrenia [38]. According to Bleuler, dissociative disorders should be considered as loosening of associations and FTD [37].

Practitioners have argued that it is the patient’s statements that constitute the picture of FTD. However, this view is not accurate. The content of verbal messages is a linguistic representation of thought processes but is not completely identical to them [4]. This fact has been highlighted by the APA, which lists among the diagnostic criteria for schizophrenia (DSM-5) precisely the disorganisation of speech, on the basis of which disorganisation of thinking can be inferred [9]. Czernikiewicz and Bibułowicz clarify the above doubts by indicating that the representations of FTD, which are the essence of schizophrenia, are disorders of the language system and cognitive deficits [39].

## 4. Materials and Methods

### 4.1. Search Strategy

A PubMed database was searched to find studies comparing levels of language and cognitive functioning in people with schizophrenia. Databases were searched from February to September 2024 using the following terms: “schizophrenia language cognition”, “schizophrenia disorganisation language”. Searches were limited to articles published in peer-reviewed journals published in English and Polish within the last 20 years (2004–2024). The initial search rendered 924 studies.

### 4.2. Exclusion Criteria

**Figure 1:**
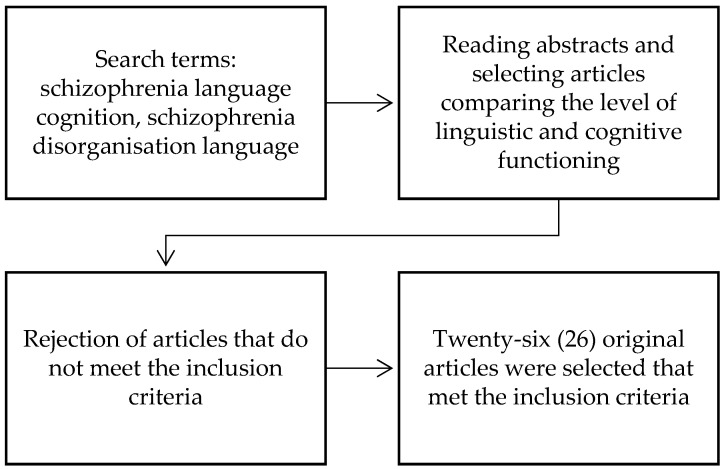
Methodological scheme.

Case studies and meta-analyses were excluded from the analyses. Also excluded were studies whose procedure for assessing linguistic behaviour deviated significantly from the definition of linguistic communication (speech) indicated in the introduction, i.e., did not involve processes of producing a coherent text organised in sound form, enabling the realisation of cognitive, communicative, and socialising activities or was not described clearly enough. Thus, analyses in which language skills were assessed only on the basis of, for example, naming objects depicted in an illustration or verbal fluency were not taken into account. Memory performance, verbal fluency, attention, and executive functions were selected for analysis of cognitive deficits because these were the areas most frequently reported as impaired in these patients. The process of methodological activities is shown in Figure 1. 

## 5. Results

The term “schizophasia”, as used in the title and subtitles, refers to the totality of speech disorders in schizophrenia regardless of their severity. Not all of the studies cited carried out a linguistic analysis to make a full diagnosis of schizophasia.

### 5.1. Schizophasia and Memory Performance

According to the authors [40], the underlying mechanism of semantic memory impairment is semantic preparation, which refers to the appropriate response to a target stimulus preceded by a baseline stimulus. The results showed that indices of perceptual preparation correlated with all phenomena from the TLC scale but that the correlation was statistically significant for only two phenomena: tangentiality and loss of goal. In a group of 27 patients in the clinical stabilisation phase, only immediate visual memory was associated with global coherence of utterances; immediate and deferred verbal memory were not associated with narrative features [41]. An assessment using the Thought and Language Disorder Scale (TALD) showed a correlation between working memory and linguistic phenomena corresponding to negative symptom syndrome, such as poverty of speech [42]. In a group of 32 patients with a diagnosis of schizophrenia, higher working memory scores were associated with, among other things, a deeper coherence of the constructed narrative and greater lexical variety. However, they did not reach statistical significance [43]. Comparing patients with a diagnosis of schizophrenia with FTD of varying severity, no significant correlation was found between working memory and TLC scale score [44].

Among patients with a diagnosis of schizophrenia or schizoaffective disorder, the association between working memory defects and language functioning scores is highlighted [45]. The results from a study of 121 patients with schizophrenia spectrum disorders (SSD) (including 89 patients with a diagnosis of schizophrenia) in the early stages of psychiatric treatment showed that a verbal memory component was associated with disorganised emotional communication and language [46].

A longitudinal study involving cyclical cognitive assessment in children who developed schizophrenia in adulthood showed consistent deficits in verbal knowledge acquisition while working memory development remained delayed (slower growth than in those without a psychiatric diagnosis) [47]. In adolescents with early-onset schizophrenia, no deficits in memory function were shown, while language domain deficits were described as subtle [48]. Among young patients with SSD, there was no statistically significant correlation between TLC score and working memory [49].

Among patients at clinically high risk of psychosis, both deficits in verbal and working memory, as well as in the language domain, were shown [50]. Analogous findings, indicating memory and language impairment as a picture of the illness, came from the results of a study assessing cognition in older people (aged 60 years and over) with schizophrenia residing in care facilities [51].

In a study assessing the language and cognitive functioning of parents of patients with SSD, which occurred in adolescence, a significant increase in the tendency for perseveration to occur in patients’ parents was shown. However, there were no differences in working memory performance between the parents of adolescent patients and the control group. Language score (TLC) correlated with working memory score in parents of adolescents with SSD. Working memory score was also associated with the number of perseverations and uncommon responses [52].

A study using magnetic resonance imaging in conjunction with an assessment performed according to TLC scale criteria showed increased resting-state cerebral blood flow in brain regions responsible for semantic processing in the temporal lobe in patients in whom phenomena from the TLC scale commonly classified as a negative dimension (e.g., blocking) were present [53]. The presence of phenomena classified as part of the negative symptom dimension was also associated with perfusion in the prefrontal lobe, whose likely functions include the operation of episodic, working, and semantic memory.

In a study of individuals from a non-clinical sample with reported elevated schizotypal features, no association was found between the presence of communication disorders and working memory performance [54].

The results are summarised in Table 1.

### 5.2. Schizophasia and Verbal Fluency

A study was conducted comparing the level of language functioning (assessed using the TLC scale) with selected cognitive functions in people with schizophrenia. Among others, verbal fluency was assessed in a variant of semantic fluency and phonemic fluency. The first variant corresponded to the task of naming as many animal names as possible, while the second variant corresponded to the task of naming as many words starting with the [k] vowel as possible. Reduced levels of verbal fluency correlated with the occurrence of individual phenomena from the TLC scale, i.e., pressure of speech, poverty of speech, circumstantiality, and echolalia. Most linguistic phenomena were negatively related to the proficiencies studied [55]. In a study by Stirling et al. [56], 30 schizophrenic patients were assessed with neurocognitive and psycholinguistic tests, which showed a negative correlation between linguistic phenomena from the TLC scale and the results of tests assessing semantic fluency. In addition, patients were divided into two groups—those showing greater or lesser deficits according to the TLC scale. The groups differed significantly in terms of semantic fluency. Higher severity of linguistic phenomena was associated with lower semantic fluency. The assessment with the TALD showed a correlation between semantic fluency and linguistic phenomena corresponding to negative symptom syndrome, e.g., concretism [42]. A linguistic analysis of Italian-speaking patients’ utterances showed no relationship between verbal fluency and the coherence of the constructed text [41].

Semantic and phonemic fluency levels were compared in geriatric patients with a diagnosis of schizophrenia. Participants with severe impairments in verbal fluency showed greater speech poverty [57].

No differences were found between the parents of patients with early-onset SSD and the control group in verbal fluency, but an increased tendency for perseveration phenomenon among the parents of patients was noted. A correlation was present between TLC score and semantic fluency in parents of SSD patients, as well as between ‘uncommon responses’ and phonemic verbal fluency [52]. The same authors investigated cognitive function and FTD (using the TLC scale) in adolescents with SSD. They found a correlation between the TLC scale score and verbal fluency impairment [49].

In a study using natural language processing techniques, no correlation was found between automatically assessed coherence and semantic and phonemic fluency [58]. In the same study, patients were also assessed using the TLC scale, where a disconnected speech index was determined using individual phenomena from the TLC scale. Analyses suggest lower consistency in verbal fluency in individuals whose speech was more disconnected. An automated analysis of the speech of people with an untreated first episode, based on an examination of conjunct use, showed that inappropriate conjunct use was not associated with verbal fluency [59].

Individuals in the non-clinical sample with reported elevated schizotypal features were categorised according to the dominant linguistic phenomena on the TLC or TLI scale—TLC disorganisation, TLC verbosity, TLC emptiness, TLI negative, and TLI idiosyncratic. Semantic fluency was significantly associated with TLC disorganisation, TLC emptiness, TLI idiosyncratic, and TLC verbosity scores. Only the association with TLC verbosity showed a positive correlation [54].

The results are summarised in Table 2.

### 5.3. Schizophasia and Attention Performance

Biondi et al. [60] presented a study comparing the magnetic resonance imaging findings of patients with a diagnosis of schizophrenia. They described connectivity within individual networks corresponding to specific anatomical structures of the brain. A positive correlation was noted between the performance of networks involved in attentional processes and a reduction in the normal flow of communication.

A study involving a linguistic analysis of 29 patients with a diagnosis of schizophrenia in the stable phase of the illness showed that reduced attentional focus is a predictor of language impairment in this illness [41]. Analyses of the utterances of 39 chronically ill patients with a diagnosis of schizophrenia showed that impaired attention affects the coherence of the resulting text. However, correlations between attentional functioning and specific types of communication failure were not statistically significant in every case [61]. Sixty patients with low severity of language phenomena were assessed by the TLC scale, and 76 patients with moderate or severe severity were compared. No statistically significant correlation was found between sustained attention and TLC scale score [44]. Similarly, no correlation was detected in the group of adolescents with SSD [49].

In a longitudinal study spanning more than 30 years, the cognitive functions of children who later developed schizophrenia were assessed cyclically. The results showed that language deficits, as an element of cognitive impairment, were present from early childhood and remained constant. However, the results of attentional functioning assessments were slightly different. Attention was found to be not only impaired, but its development over the years was slower than that observed in the control group [47]. In adolescents with early-onset schizophrenia, attentional processes were reported to be more disturbed than language functioning [48].

Patients at clinically high risk of psychosis showed the presence of both attention and language disorders [50].

Docherty et al. [45] investigated the processes underlying communication failures in 63 patients with a diagnosis of schizophrenia or schizoaffective disorder. Their findings align with the data presented by the authors, showing that most measures of attention were associated with scores on a scale assessing speech impairment.

In the group of individuals with reported elevated schizotypal traits, attentional performance was only associated with the part of the TLI scale score corresponding to language behaviour attributed to the negative syndrome [54].

The results are summarised in Table 3.

### 5.4. Schizophasia and Executive Function

A correlation was identified between language disorders, as assessed using the TLC scale, and the ability to think abstractly in individuals with schizophrenia. Additionally, language disorders were correlated with several indicators from executive function tests. The ability to formulate logical concepts was evaluated, and patients experiencing challenges in this area were more likely to exhibit language impairments [55]. A study comparing patients with schizophrenia who had varying severities of FTD, assessed using the TLC scale, demonstrated that higher problem-solving abilities were linked to lower rates of FTD. A correlation was also observed between negative phenomena from the TLC scale and deficits in reasoning and problem-solving [44]. Assessment of FTD with the TALD scale, which also encompasses linguistic phenomena, revealed an association between positive FTD and deficits in executive function [42]. In a separate comparison of cognitive and linguistic functioning using linguistic analysis conducted with 27 clinically stable patients with schizophrenia, executive function deficits were identified as predictors of language deficits [41]. However, in a group of 32 individuals diagnosed with schizophrenia, greater narrative coherence did not correspond to higher executive functioning [43].

A study comparing patients with first-episode schizophrenia and healthy controls found that low scores for conceptual organisation, understood by the authors as impaired speech, were associated with poor analytical thinking [62]. An intriguing study by Silva et al. [63] assessed conceptual disorganisation and analytical thinking in patients experiencing their first episode of schizophrenia. Using specialised software, the study analysed linguistic style dominance, distinguishing between categorical (formal, hierarchical thinking patterns) and narrative (intuitive, episodic thinking patterns) styles. The findings indicated a reduced use of categorical language style in patients with first-episode schizophrenia, which the authors interpreted as evidence of diminished analytical thinking compared to controls. Lower analytical thinking scores were linked to higher levels of conceptual disorganisation and disorganised thinking. Furthermore, language style emerged as a stronger predictor of conceptual disorganisation than cognitive measures of processing speed.

In adolescents with early-onset schizophrenia, executive function disruptions were more pronounced than language impairments [48]. Similarly, deficits in executive and language functions are observed in individuals at clinically high risk for psychosis [50].

A longitudinal study assessing cognitive development in children who later developed schizophrenia into adulthood found early and consistent deficits in vocabulary acquisition. The vocabulary subtest measured the ability to understand word meanings, recall them, and construct coherent and effective speech. In contrast, the development of visuospatial problem-solving skills was delayed, progressing more slowly than in the control group [47].

A study comparing the executive functioning of parents of adolescents with SSD to a control group found no significant differences. However, an increased tendency for perseveration was noted among the parents of SSD patients [52]. Additionally, the same authors identified a correlation between executive function deficits and TLC scale scores in adolescents with SSD [49].

Magnetic resonance imaging studies revealed that formal thought disorders, as assessed by the TLC scale, are associated with structural abnormalities in brain regions responsible for language and executive functions. These areas include the left superior temporal lobe, overlapping with Wernicke’s area, and the left inferior operculum, overlapping with Broca’s area [64].

Studies comparing the cognitive and linguistic deficits of individuals with schizophrenia to those with aphasia revealed a strong link between executive function impairments and formal thought disorders, particularly conceptual disorganisation [65].

Among individuals with elevated schizotypal traits, a connection was observed between language impairments (as assessed using the TLC and TLI scales) and executive functions. These impairments, alongside higher-order reasoning deficits, were identified as key drivers of FTD in this population [54].

The results are summarized in Table 4.

## 6. Discussion

This summary provides a comprehensive review of the literature on cognitive and language deficits in schizophrenia. Among the most frequently mentioned cognitive impairments are deficits in memory, attention, executive functions, and verbal fluency. Consequently, this review focuses on studies comparing these specific skills with the language assessments of individuals with schizophrenia. Clinical studies have often aimed to identify relationships between the performance of these cognitive functions and individual phenomena in language assessments. The cited research reveals varying conclusions. Some studies found significant correlations between cognitive functions and specific language components [40,41,42,44,45,46,47,51,55,56,57,61,63,65], while others did not report statistically significant correlations [43,48]. Partial correlations were observed in adolescents with SSD [49], patients at clinically high risk of psychosis [50], and the parents of adolescent patients, albeit only for certain measures [52]. In individuals with increased schizotypal traits, memory performance did not correlate with language deficits, though links were observed between language deficits, verbal fluency, and executive functions. Attention was only associated with specific language phenomena [54]. Automated speech analysis confirmed [58] and refuted [59] relationships between language function and cognitive deficits. Neuroimaging studies generally supported a relationship between language and cognitive functions in schizophrenia [53,60,62,64].

Most of the analysed studies confirm the relationship between language and cognitive impairment in schizophrenia. In most results, however, this relationship concerns individual components of linguistic or cognitive assessment. Similar conclusions are shown by other meta-analyses comparing the linguistic and cognitive functioning of people suffering from schizophrenia. According to analyses, patients suffering from schizophrenia are characterised by a reduction in the efficiency of all cognitive domains, including language [66,67,68]. Scientists indicate that neurocognitive and linguistic correlates of (positive or negative) FTD might be partly different [69]. Reviews assessing the effects of cognitive training and transcranial direct current stimulation in neuropsychiatric disorders show non-statistically significant effects favouring combined treatment on global cognition and language [70]. Comparing neuroimaging studies proved that abnormal activation in brain regions that are associated with language and speech processing extends to higher-order cognitive functions [71].

The methodological differences associated with the use of different instruments represent an important limitation in attempting to compare the above results. Although some of the phenomena are common and are part of the assessment in most of the referenced scales, seemingly minor differences may prove crucial in a comparative diagnosis. The TLC scale includes 18 language phenomena, whereas the TALD scale, based on other scales, including the TLC, includes a total of 30 such phenomena. As both scales refer to the same phenomena, one phenomenon on the TLC scale may correspond to several phenomena on the TALD scale. For example, the phenomenon of pressure of speech on the TLC scale corresponds to the phenomenon of pressured speech on the TALD scale, but it also corresponds to the phenomenon of logorrhoea. Therefore, on the TALD scale, the phenomenon is separated from the TLC scale into two phenomena that need to be differentiated from each other. This results in a situation where the determination of the severity of a particular phenomenon on the TLC scale (on a numerical scale) may be split into scores for two separate phenomena on the TALD scale, making it significantly more difficult to compare these scores with each other. On the TALD scale, there is additionally an element of subjective patient assessment, which is not included in other scales. The opposite is true for the CDI scale, which indicates fewer, i.e., only six types of communication failure in conversational speech. As there are far fewer categories, they cover a wider range of phenomena, and some of the phenomena from the TLC or TALD scales are not referenced there, such as pressure of speech or loss of goal. The same is true of the SANS/SAPS or TLI scales, which also encompass fewer categories than the most commonly applied TLC scale. In the case of the PANSS scale, behaviours corresponding to the TLC-scale linguistic phenomena have been defined very broadly, and many of the TLC-scale phenomena comprise a single PANSS scale item, making it significantly more difficult to adequately compare results between the two scales. In order to minimise the effects of this limitation, it would seem helpful if reports of language pathology were also supported by linguistic assessment. Most of the assessed phenomena are subject to the subjective assessment of the researcher and, even despite the detailed instructions of the authors of the mentioned scales, smaller or larger differences may appear between researchers regarding the assessment of the severity of particular phenomena. The linguistic rules of speech are fixed for each language, and, by definition, their assessment may proceed in a more objective manner. However, this does not imply that the assessment of grammatical knowledge offers a complete picture of the level of linguistic functioning. A study assessing language and cognitive function in patients with a diagnosis of schizophrenia and patients with aphasia [65] used the TROG-2, which assesses comprehension of grammatical structures (of the English language). In schizophrenia, the understanding of grammatical structures and even their correct use does not necessarily imply the absence of language pathology in individual patients. The correct grammatical structure of sentences may be preserved in the utterances of patients with schizophrenia. Yet, their utterances may be completely unintelligible due to pragmatic incoherence, a phenomenon that may be identified by TLC-scale phenomena, e.g., tangentiality, incoherence, illogicality, circumstantiality, loss of goal, stilted speech. However, since some of the phenomena identified above as prone to error by subjective assessment concern sentence structure and word choice (syntax and semantics), linguistic assessment could support clinical evaluation. Perhaps combining elements of clinical and linguistic assessment would yield the most complete picture of the linguistic functioning of schizophrenic patients.

Analogous limitations apply to natural language processing techniques. One study utilised Latent Semantic Analysis (LSA) [58], a method that demonstrated effectiveness in evaluating the semantic consistency of utterances in individuals with schizophrenia. However, researchers have pointed out a significant limitation: the sensitivity of this method in distinguishing between schizophrenic patients and healthy controls depends heavily on the type of question posed [72]. Moreover, automated analysis cannot assess the situational reality or contextual relevance of an utterance. As noted earlier, syntactic and semantic coherence alone does not guarantee communicative integrity. For instance, poverty of content of speech involves linguistically coherent sentences that lack substantive content. Similarly, in distractible speech, utterances are interrupted and redirected by external environmental stimuli. While automated techniques might detect inconsistency in such cases, identifying whether this behaviour stems from environmental distraction or distorted semantic pathways requires observing the patient as they construct the utterance. Another study employed the Coh-Metrix instrument, which evaluates deep cohesion in addition to semantic coherence. This approach provides insights into functional communication beyond semantic relationships alone [43]. Nevertheless, it remains constrained by its inability to interpret non-verbal cues, a crucial component of communicative meaning.

Studies comparing language and cognitive functioning in schizophrenia often face significant inconsistencies in terminology. A notable challenge relates to differences in the meanings of the terms ‘speech disorder’ and ‘thought disorder.’ Although the APA has clarified these definitions—identifying disorganised speech as a symptom of schizophrenia, from which disorganised thinking can be inferred [9]—many researchers still use these terms interchangeably. However, since speech disorders are empirically assessable, some researchers argue that the term ‘speech disorder’ is more appropriate, given the inherent difficulty of directly assessing ‘thinking’ without relying on speech as an indicator [36]. The TLC scale, commonly used in the cited studies, assesses thought, language, and communication. The phenomena it measures pertain to linguistic behaviours, making it a valuable tool for both clinical and linguistic research. Nevertheless, the overlap between speech and thought deficits, assessed through essentially the same symptom (speech disorder), complicates interpretations of their relationships with other schizophrenia symptoms. In some analyses, speech and thought deficits are compared using different scales that ultimately evaluate the same linguistic skills. It is important to note that in most of the cited studies using the TLC scale (as shown in Table 1, Table 2, Table 3 and Table 4), the authors explicitly stated that they were assessing formal thought disorders with this tool.

Another issue seen in contemporary analyses is the challenge of studying speech disorders in schizophrenia. Linguists have developed tools for assessing schizophasia, such as the SSRS (Short Schizophasia Rating Scale (pol. KSOS—Krótka Skala Oceny Schizofazji)) [73], while clinicians have multiple tools, including the TLC, TLI, and TALD scales. However, the primary difficulty does not lie in the choice of tool but rather in the selection of appropriate tasks. The key consideration is what aspect of speech or language is intended to be examined. Assuming a focus on linguistic (speech) assessment, tasks must be designed to involve genuine linguistic communication, the definition and components of which were outlined in the introduction. Linguistic communication entails the sharing of knowledge with the recipient to achieve socialisation goals. Therefore, tasks such as naming objects in an illustration do not provide a full representation of linguistic communication, as they fail to assess communicative competence. Communicative competence is defined as the ability to adapt one’s speech to ensure it is comprehensible to the recipient [2]. Studies by linguists have shown that in schizophasia, it is the communicative function of language that deteriorates [12]. Thus, to assess formal thought disorders—deduced from the presence of speech disorders—it is necessary to investigate linguistic communication in its entirety. Suitable tasks for this purpose include instructing participants to construct a narrative or provide a description, such as recounting their childhood or describing a presented image [4]. This review represents the first attempt to address the definitional difficulties in understanding what formal thinking disorders are, with strict inclusion criteria based on language assessment. Other reviews and meta-analyses known to the authors comparing cognitive functioning in people diagnosed with schizophrenia presenting with language deficits/formal thinking disorders have included studies based on the assessment of formal thinking disorders in their analyses without specifying exactly what assessment criteria the study must include to be considered to adequately examine the selected ability. Studies where linguistic assessment did not involve the construction of a coherent text for communicative purposes were excluded from this study; for example, tasks of naming illustrations or verbal fluency only were excluded as linguistic assessment. Not all researchers stressed that in order to assess formal thought disorders, it is also essential to include a task in which the participant builds a longer utterance addressed to a specific audience; therefore, one may not be certain about the conditions under which FTD assessments are made. Some researchers [69] have even stressed that they do not include studies assessing communication disorders in their analyses, although, according to the knowledge promoted by the APA, it is on the basis of linguistic behaviour that the presence of FTD is inferred. Thus, the present analysis makes (as far as we know) the first attempt to compare studies assessing cognitive dysfunction with linguistic dysfunction, taking into account a critical evaluation of the modality in which speech disorders were assessed in the individual studies.

Perhaps an important issue to consider is the definition of speech itself. According to the definition presented earlier, speech serves crucial communicative and socialising functions, which are not always fully accounted for in research. Speech is not merely the random production of words but rather a structured process that enables effective communication. One study assessed formal thought disorders (using the TLC scale) and semantic coherence through an automated evaluation of communicative abnormalities [58]. Formal thought disorders were analysed based on several minutes of patient conversations, whereas the automated language analysis component focused on speech produced during a verbal fluency task. While the relationship between the semantic coherence of items mentioned in the fluency task is undoubtedly valuable, the study of formal thought disorders assessed what truly constitutes ‘speech,’ particularly considering its essential socialising function. Studies show that patients with high levels of schizophasia have a reduced level of psychosocial functioning. It is assumed that communication deficits hinder social functioning [74], which is an extremely important factor from the perspective of the rehabilitation of psychiatric patients.

## 7. Conclusions

Both linguistic and clinical studies confirm that language disorders are integral to the presentation of schizophrenia. Linguistic analyses conducted by Polish researchers provide precise descriptions of the linguistic phenomena observed in patients with schizophrenia. These findings are corroborated by studies conducted among patients who speak other languages, demonstrating the universality of these phenomena. However, studies juxtaposing linguistic and cognitive functioning in schizophrenia are predominantly conducted by clinicians.

There is a scarcity of research that comprehensively examines the linguistic functioning of individuals with schizophrenia and compares it with their cognitive functioning. Studies by clinicians frequently rely on the TLC scale, which serves primarily as an index of schizophasic phenomena [75]. While the scale is effective in naming observed phenomena and determining their severity, it does not enable a comprehensive linguistic diagnosis. For instance, it does not assess the complexity of syntax in patient utterances, a factor known to be impoverished in schizophrenia [13,14]. This limitation is particularly significant when comparing linguistic deficits with analogous cognitive abilities, such as executive functions (e.g., planning), which could provide valuable insights.

The studies cited above yield varied results. Researchers have both confirmed and dismissed relationships between linguistic functioning and specific cognitive functions. In some cases, correlations are observed but fail to reach statistical significance. Nonetheless, the majority of the studies indicate correlations of varying degrees between linguistic and cognitive functioning in schizophrenia. This inconsistency suggests that correlations may or may not be present in individual patients. Future research should aim to identify the common characteristics of patients in whom such correlations are evident. Given the predictive importance of cognitive deficits in schizophrenia, linking descriptions of cognitive functioning during the prodromal phase with linguistic behaviours associated with specific symptoms could have significant scientific and practical implications. Such research could aid in the early detection of high-risk psychosis states, enhancing our understanding of the illness and potentially improving early intervention strategies.

## Figures and Tables

**Table 1:** Schizophasia and memory performance.

Author	Year of Publication	Study Group	Number of Participants	Language of Respondents	Language Assessment Tool	Method of Cognitive Assessment	Main Findings
Quelen et al. [40]	2005	Schizophrenia	20	French	TLC	Guessing words based on semantic cues (as a mechanism underlying the functioning of semantic memory)	Indicators of perceptual preparedness are significantly correlated with the phenomenon of tangentiality and loss of goal.
Marini et al. [41]	2008	Schizophrenia	27	Italian	Verbal productivity, lexical and morpho-syntactic organisation, informativeness, and textual organisation of the resulting narrative were evaluated	Rey’s 15-word Immediate Recall, Delayed Recall, Immediate Visual Memory	No statistically significant correlation.
Mutlu et al. [42]	2021	Schizophrenia	46	Turkish	TALD	ACT	Significant relationship between working memory and negative syndrome TALD scores.
Lundin et al. [43]	2023	Schizophrenia	32	English	Coh-Metrix 3.0	MCCB	Higher working memory scores are associated with greater lexical consistency and variety (no statistical significance).
Comparelli et al. [44]	2020	Schizophrenia	136	Italian	TLC	Corsi block test: spatial span, TMT B-A subtest	No statistically significant correlation.
Docherty et al. [45]	2013	Schizophrenia or schizoaffective disorder	63	English	CDI	DST	The relationship between working memory deficits and speech disorders.
Skiba et al. [46]	2024	Schizophrenia, schizoaffective disorder or schizo-like disorder	121	English	SANS, SAPS	DST, Logical Memory, Spatial Span, Visual Reproduction	Relationship between verbal memory score and impoverished and disorganised communication.
Reichenberg et al. [47]	2010	Children who later developed schizophrenia (longitudinal study)	35	English	WISC-R	WISC-R	Developmental delay in working memory, static deficits in the “vocabulary” test.
Rhinewine et al. [48]	2005	Adolescents with early-onset schizophrenia	54	English	Controlled Word Association Test (letter), WISC/WAIS-III	Child/Adult Edition CVLT total trials 1–5, CVLT delay-free recall	No memory deficits, subtle relative sparing of language function.
Remberk et al. [49]	2012	Early-onset SSD	32	Polish	TLC, KRT	DST Backward	No statistically significant correlation.
Carrión et al. [50]	2011	Clinical high risk for psychosis	127	English	WISC-III, WAIS-R Wide-Range Achievement Test 3, BNT	WISC-III, WISC-R, CVLT, Wechsler Memory Scale	Significant deficits in the domains of verbal memory, working memory, and language.
Diaz et al. [51]	2011	Schizophrenia (patients with late-life)	10	Portuguese	CAMCOG	CAMCOG, CAMDEX	Significant lower scores on language and memory tests than the control group.
Remberk et al. [52]	2012	Parents of SSD adolescents	38	Polish	TLC, KRT	DST Backward	Correlation between TLC score and working memory, as well as between working memory score and number of perseverations and uncommon responses.
Stegmayer et al. [53]	2017	SSD	47	German	TLC	- MRI	Negative phenomena from the TLC scale are associated with perfusion in the precuneus.
Deyo et al. [54]	2021	Non-clinical sample with elevated schizotypal traits	91	English	TLC, TLI	CANTAB, CVLT-II	No relationship between linguistic phenomena and memory.

Note: The table indicates only those tools or methods related to the skills that are the subject of this list. TLC—the Scale for the Assessment of Thought, Language and Communication; TALD—Thought and Language Disorder Scale; ACT—Auditory Consonant Trigram Test; MCCB—MATRICS Consensus Cognitive Battery; CDI—Communication Disturbances Index; TMT—Trail Making Test; DST—Digit Span Test; SANS/SAPS—The Scale for Assessment of Negative/Positive Symptoms; WISC-R—Wechsler Intelligence Scale for Children—Revised; WISC—Wechsler Intelligence Scale for Children; WAIS—Wechsler Adult Intelligence Scale; CVLT—California Verbal Learning Test; SSD—schizophrenia spectrum disorder; KRT—Kent–Rosanoff Test; BNT—Boston Naming Test; CAMCOG—Cambridge Cognition Examination; CAMDEX—The Cambridge Examination for Mental Disorders of the Elderly; “-“—not assessed on the basis of tests; MRI—magnetic resonance imaging; TLI—Thought and Language Index; CANTAB—Cambridge Neuropsychological Test Automated Battery.

**Table 2:** Schizophasia and verbal fluency.

Author	Year of Publication	Study Group	Number of Participants	Language of Respondents	Language Assessment Tool	Method of Cognitive Assessment	Main Findings
Waszkiewicz et al. [55]	2012	Schizophrenia	45	Polish	TLC	SFT, PFT	Lower levels of verbal fluency correlate with pressure of speech, poverty of speech, circumstantiality, and echolalia.
Stirling et al. [56]	2006	Schizophrenia	30	English	TLC	GNT, PPT, SFT and semantic efficiency, QT	GNT, QT, and semantic fluency correlate with TLC score. Semantic fluency predicts TLC scores.
Mutlu et al. [42]	2021	Schizophrenia	46	Turkish	TALD	SFT, PFT, Alternation	Significant relationship between semantic fluency and negative syndrome TALD scores.
Marini et al. [41]	2008	Schizophrenia	27	Italian	Verbal productivity, lexical and morpho-syntactic organisation, informativeness, and textual organisation of the resulting narrative were evaluated	SFT, PFT	No statistically significant correlations between the tested abilities.
Bowie et al. [57]	2004	Schizophrenia (age ≥ 50)	392	English	TLC	SFT, PFT	Higher poverty of speech in patients with lower verbal fluency scores.
Remberk et al. [52]	2012	Parents of SSD adolescents	38	Polish	TLC, KRT	SFT, PFT	The relationship between TLC score and verbal fluency. Uncommon responses related to PFT.
Remberk et al. [49]	2012	Early-onset SSD	32	Polish	TLC, KRT	SFT, PFT	TLC test negatively correlated with verbal fluency test results. No significant correlation between verbal fluency tests and KRT results.
Holshausen et al. [58]	2014	Schizophrenia (age ≥ 50)	165	English	TLC, LSA	SFT, PFT	Average vector length (a measure of unusualness of words), determining the representation of semantic relations between words, significantly related to semantic and phonemic fluency and disconnectedness in speech (assessed using the TLC scale).
Mackinley et al. [59]	2021	FEP, acute phase of illness	39	English	TLI, Coh-Metrix 3.0	SFT	No significant correlation between semantic fluency and the use of connectives.
Deyo et al. [54]	2021	Non-clinical sample with elevated schizotypal traits	91	English	TLC, TLI	D-KEFS Category Fluency	Significant negative correlation between TLC disorganisation, TLC emptiness, TLI idiosyncratic, and verbal fluency. TLC verbosity significantly correlated with verbal fluency.

FEP—first episode of psychosis; SFT—Semantic Fluency Task; PFT—Phonological Fluency Task; GNT—Graded Naming Test; PPT—The Pyramids and Palm Trees Test; QT—The Quick Test; TALD—Thought and Language Disorder Scale; LSA—Latent Semantic Analysis.

**Table 3:** Schizophasia and attention performance.

Author	Year of Publication	Study Group	Number of Participants	Language of Respondents	Language Assessment Tool	Method of Cognitive Assessment	Main Findings
Biondi et al. [60]	2024	Schizophrenia	74	No data available, probably different	PANSS, fMRI	PANNS, fMRI	The higher the connectivity of the networks involved in attentional processes, the lower the spontaneity and flow of the conversation.
Marini et al. [41]	2008	Schizophrenia	27	Italian	Verbal productivity, lexical and morpho-syntactic organisation, informativeness and textual organisation of the resulting narrative were evaluated	TMT A, B	The score of the attention assessment subtests was the only predictor of global consistency errors.
Docherty et al. [61]	2006	Chronic schizophrenia	39	English	CDI	The test of sustained attention	A negative correlation was found between communication failures and attention test scores. For some types of communication failures, the correlations with attention functioning were not statistically significant.
Comparelli et al. [44]	2020	Schizophrenia	136	Italian	TLC	WCST (by counting the number of nonperseverative errors)	No statistically significant correlation.
Remberk et al. [49]	2012	Early-onset SSD	32	Polish	TLC, KRT	DST Forward	No statistically significant correlation.
Reichenberg et al. [47]	2010	Children who later developed schizophrenia (longitudinal study)	35	English	WISC-R	WISC-R	Developmental deficits in the acquisition of verbal knowledge, as well as developmental delays in the area of attention in children who have developed schizophrenia in adulthood.
Rhinewine et al. [48]	2005	Adolescents with early-onset schizophrenia	54	English	Controlled Word Association Test (letter), WISC/WAIS-III	WISC/WAIS-III, TMT A, CPT-IP	Subtle relative sparing of language function. Linguistic domain significantly less impaired than attention.
Carrión et al. [50]	2011	Clinical high risk for psychosis	127	English	WISC-III, WAIS-R, Wide-Range Achievement Test 3, BNT	CPT-IP Version, 2-, 3-, and 4-digit	Significantly impaired language functioning and attention processes in people at clinically high risk for psychosis.
Docherty et al. [45]	2013	Schizophrenia or schizoaffective disorder	63	English	CDI	CPT-IP	Significant correlation between linguistic functioning and most measures of attention.
Deyo et al. [54]	2021	Non-clinical sample with elevated schizotypal traits	91	English	TLC, TLI	DST Forward, CVLT-II-Trial 1, CANTAB Rapid Visual Information Processing, ADSDT-distractible proportion, ADSDT-distractible intrusions	Higher TLI Negative associated with lower performance on attention span and sustained attention.

PANSS—Positive and Negative Syndrome Scale; fMRI—functional magnetic resonance imaging; WCST—Wisconsin Card Sorting Test; CPT-IP—Continuous Performance Test, Identical Pairs Version; ADSDT—Auditory Digit Span Distraction Test.

**Table 4:** Schizophasia and executive function.

Author	Year of Publication	Study Group	Number of Participants	Language of Respondents	Language Assessment Tool	Method of Cognitive Assessment	Main Findings
Waszkiewicz et al. [55]	2012	Schizophrenia	45	Polish	TLC	WCST	Language deficits correlate with some measures of executive function.
Comparelli et al. [44]	2020	Schizophrenia	136	Italian	TLC	N5 PANSS, WCST	Negative linguistic phenomena correlate with reasoning and problem solving.
Mutlu et al. [42]	2021	Schizophrenia	46	Turkish	TALD	TMT B	Correlation between positive factors of TALD scores and executive functions.
Marini et al. [41]	2008	Schizophrenia	27	Italian	Verbal productivity, lexical and morpho-syntactic organisation, informativeness, and textual organisation of the resulting narrative were evaluated	WCST	Number of non-perseverative errors in WCST was a predictor of % paragrammatic errors.
Lundin et al. [43]	2023	Schizophrenia	32	English	Coh-Metrix 3.0	TMT B	No statistically significant correlation.
Limongi et al. [62]	2023	FES	30	English	TLI	- fMRI (at rest)	Low conceptual organisation results in low analytical thinking.
Silva et al. [63]	2021	FES	78	English	TLI, PANSS	LIWC 2015 Edition	Less categorical linguistic style in the FES group than in health control. The higher the conceptual disorganisation, the lower the analytical thinking.
Rhinewine et al. [48]	2005	Adolescents with early-onset schizophrenia	54	English	Controlled Word Association Test (letter), WISC/WAIS-III	WCST, TMT B	Executive functions significantly more impaired than the language domain.
Carrión et al. [50]	2011	Clinical high risk for psychosis	127	English	WISC-III, WAIS-R Wide-Range Achievement Test 3, BNT	Ruff Figular Fluency Test, WCST version 2	Significant impairment of executive functions. Significant language deficits also present, but at a lower level.
Reichenberg et al. [47]	2010	Children who later developed schizophrenia (longitudinal study)	35	English	WISC-R	WISC-R	Language deficits and reasoning deficits at a constant level throughout adolescence. Delayed development of visual-spatial problem-solving skills.
Remberk et al. [52]	2012	Parents of SSD adolescents	38	Polish	TLC, KRT	WCST	No differences between the group of parents of SSD patients and the control group.
Remberk et al. [49]	2012	Early-onset SSD	32	Polish	TLC, KRT	WCST	Correlation of TLC results and number of non-perseverative errors in WCST.
Sans-Sansa et al. [64]	2013	Schizophrenia	51	Spanish	TLC	- MRI	The TLC score correlated with a reduction in the volume of brain structures responsible for language and executive functions.
Little et al. [65]	2019	Schizophrenia/Aphasia	30/20	English	TROG-2, BNT, WASI-II, PANSS	Block Design, Matrix Reasoning, BSAT	Largest language deficits in patients with aphasia, smaller in SZ+FTD, smallest in SZ-FTD. Greater executive function deficits in SZ+FTD than in SZ-FTD. Correlation of verbal and nonverbal scores only in patients with schizophrenia.
Deyo et al. [54]	2021	Non-clinical sample with elevated schizotypal traits	91	English	TLC, TLI	CANTAB SOC, CANTAB IED, Reality Monitoring Task, among others.	Higher TLI scores, as well as portions of TLC scores, are associated with poorer executive function scores. Higher TLC Verbosity scores are associated with better cognitive performance.

FES—first episode of schizophrenia; LIWC—Linguistic Inquiry Word Count; TROG-2—The Test for Reception of Grammar; WASI—Wechsler Abbreviated Scale of Intelligence; BSAT—The Brixton Spatial Anticipation Test; SZ—schizophrenia; SOC—Stockings of Cambridge; IED—Intra-extra Dimensional Set Shift.
